# Transcriptome and expression profiling analysis link patterns of gene expression to antennal responses in *Spodoptera litura*

**DOI:** 10.1186/s12864-015-1375-x

**Published:** 2015-04-07

**Authors:** Bo Feng, Xinda Lin, Kaidi Zheng, Kai Qian, Yongchang Chang, Yongjun Du

**Affiliations:** Institute of Health and Environmental Ecology, Wenzhou Medical University, University Town, Wenzhou, 325035 China; College of Life Sciences, China Jiliang University, Hangzhou, Zhejiang 310018 China; Division of Neurobiology, Barrow Neurological Institute, St. Joseph’s Hospital and Medical Center, Phoenix, AZ 85013 USA

**Keywords:** Olfactory receptor, RT-qPCR, Electroantennogram, Sex-biased expression, Sex pheromone, Plant volatiles

## Abstract

**Background:**

The study of olfaction is key to understanding the interaction of insects with their environment and provides opportunities to develop novel tactics for control of pest species. Recent developments in transcriptomic approaches enable the molecular basis of olfaction to be studied even in species with limited genomic information. Here we use transcriptome and expression profiling analysis to characterize the antennal transcriptome of the noctuid moth and polyphagous pest *Spodoptera litura*.

**Results:**

We identify 74 candidate genes involved in odor detection and recognition, encoding 26 ORs, 21 OBPs, 18 CSPs and 9 IRs. We examine their expression levels in both sexes and seek evidence for their function by relating their expression with levels of EAG response in male and female antennae to 58 host and non-host plant volatiles and sex pheromone components. The majority of olfactory genes showed sex-biased expression, usually male-biased in ORs. A link between OR gene expression and antennal responses to odors was evident, a third of the compounds tested evoking a sex-biased response, in every case also male-biased. Two candidate pheromone receptors, *OR14* and *OR23* were especially strongly expressed and male-biased and we suggest that these may respond to the two female sex pheromone components of *S. litura*, Z9E11-14:OAc and Z9E12-14:OAc, which evoked strongly male-biased EAG responses.

**Conclusions:**

Our results provide the molecular basis for elucidating the olfactory profile of moths and the sexual divergence of their behavior and could enable the targeting of particular genes, and behaviors for pest management.

**Electronic supplementary material:**

The online version of this article (doi:10.1186/s12864-015-1375-x) contains supplementary material, which is available to authorized users.

## Background

Olfaction plays a key role in the interactions of insects with their environment, mediating foraging, aggregation, mating, and oviposition behaviors. Studies of insect olfaction have provided fundamental insights into chemosensory biology and chemical ecology [1-4] and have presented valuable opportunities for pest management [5-8]. Lepidoptera are a focus of interest for studies of olfaction as they have large and sensitive olfactory repertoires [8] yet molecular studies of olfaction in Lepidoptera lag behind those in standard insect models. Recently, there has been exciting progress in identifying genes coding for lepidopteran olfactory receptors, not only in the model *Bombyx mori* [8-10], for which there is genomic data [11], but also in the pest species *Manduca sexta* [12], *Heliothis virescens* [13,14] and *Spodoptera frugiperda* [15] , however it’s a draft assembly to present, which would be a better reference for *S.litura* in the future. Progress in the absence of genomic data has been made possible by genome-wide approaches for transcriptome analysis, such as RNA-Sequencing (RNA-Seq) [12]. Such high-throughput molecular techniques and associated informatics technologies, are becoming commonplace in chemical ecology [9,16,17].

A sufficient level of expression of genes is key to the success of transcriptomic approaches to their identification. In an elegant and comprehensive study on the antennal transcriptome of *M. sexta*, Grosse-Wilde *et al.* [12] identified the main olfactory genes and compared their expression in males and females. In an equally thorough study of the antennal transcriptome of *S. littoralis*, Jacquin-Joly *et al.* [18,19] examined the expression of 7 olfactory and 4 gustatory receptors in different tissues and discussed their function. They suggested that transcriptome expression may change following mating and could reveal more olfactory genes involved in sex-specific behavior. If expression levels of olfactory genes could be linked with functional responses to volatiles, expression profiling could lead to a better understanding of the function and operation of olfactory genes and could elucidate how individual variation of olfactory gene expression might lead to speciation or resistance to pheromonal pest management.

Olfactory neurons express many proteins involved in the capture of volatiles from the environment and signal transduction. These include olfactory receptors (ORs) [14,19-21], odorant-binding proteins (OBPs) [22,23], chemosensory proteins (CSPs) [23,24] and ionotropic receptors (IRs) [25]. ORs specifically bind odorant molecules and initiate signal transduction in the membrane of the olfactory neuron. Insect ORs generally exhibit low levels of homology and are selectively expressed in olfactory neurons at low levels [26]. Members of the OR83b receptor subfamily, commonly known as olfactory receptor coreceptors (ORCOs), are more conserved and expressed in most olfactory neurons at various stages of development [27]. In Lepidoptera, OBPs are classified into pheromone-binding proteins (PBPs) [28], general odorant-binding proteins (GOBPs) [29,30] and antennal-binding proteins (ABPs) [31]. ABPs are expressed specifically in the antenna with characteristics typical of OBPs [29,32,33] but they have low homology with PBPs and GOBPs and their function remains unknown. The CSPs constitute a conserved family of binding proteins that are unrelated to OBPs and whose function is again unclear [34,35]. IRs, recently described novel family of olfactory receptors [36], are localized on the dendrite of chemosensory neurons and are ligand-gated ion channels that mediate chemical communication between neurons [37]. IRs were further classified into two sub-families: conserved “antennal IRs” involved in olfaction and species-specific “divergent IRs” that might be associated with gustation [37,38].

The tobacco cutworm moth, *Spodoptera litura,* is an important agriculture pest widely distributed throughout tropical and temperate Asia, Australia and the Pacific Islands (Additional file 1: Figure S1), noctuid moth and a polyphagous pest with more than 290 host plants belonging to 99 families [39]. The *S. litura* attacks numerous economically important crops and trees, it also defoliated these crops or trees, finally leads to serious economic yield loss [40]. Its two-component sex pheromone has been identified [41], together with a plant-derived synergist [42], and the pheromone is used at a large scale for mass trapping for pest management [42]. However, little is known about *S. litura*’s ORs, CSPs and OBPs [43-46]. Previous behavioral bioassays have demonstrated that male and female moths respond differently to odorants, including pheromones [41]. The complete genome of *S. litura* is not yet available.

Here we study the molecular mechanisms underlying sex-specific response to odors, including sex pheromones, in *S. litura*, and discuss the link between OR gene expression and chemosensory responses as measured by electroantennography. Using *de novo* transcriptome and expression profile analysis we achieved a high level of coverage of olfactory genes and measured gene expression using both single-end RNA-Seq and RT-qPCR to give confidence in comparisons between sexes at lower expression levels. We discuss the implications of our study for understanding the functioning of olfactory genes.

## Results

### Olfactory responses of male and female moths

**Figure 1:**
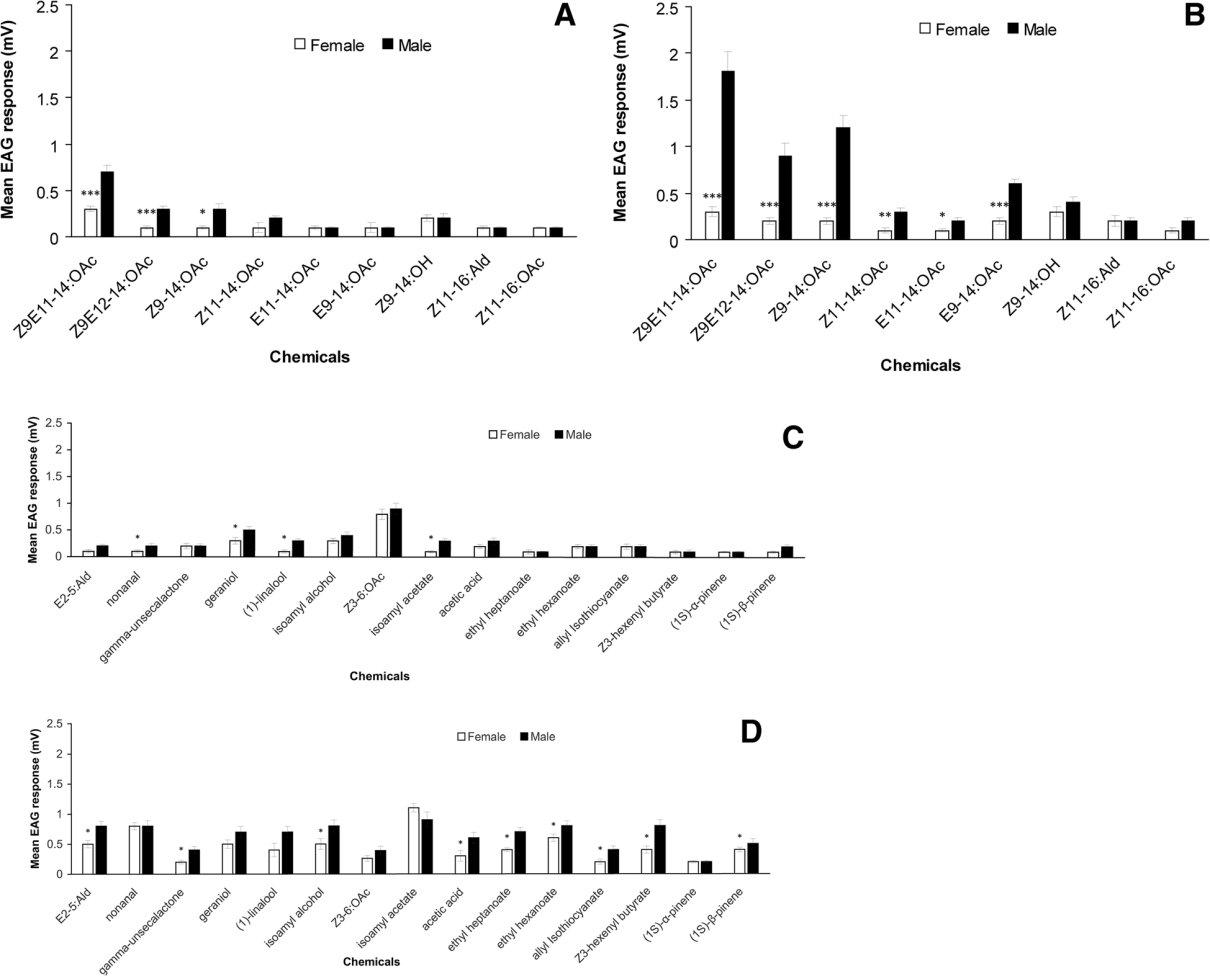
**Electroantennogram responses recorded from male and female** ***S. litura*** **antennae elicited by: A and B sex pheromones and their isomers; C and D floral scents and plant volatiles. A** and **C** stimulating dosage 10^−4^ v/v, **B** and **D** stimulating dosage 10^−2^ v/v, see Methods and materials for details. Error bars signify SEM. Significance of difference between male and female responses indicated by *P < 0.05, **P < 0.01, ***P < 0.001, Students *t* test.

The antennae of both sexes showed varying electroantennogram (EAG) responses to the 58 chemicals that were presented, representing flower volatiles, host or non-host plant volatiles, and *S. litura* sex pheromone components and their isomers (Figure 1 and Additional file 1: Figure S2). For 11 floral odors, two plant volatiles, and six sex pheromone components or isomers EAG responses differed significantly between sexes, and in each case males responded more strongly (Figure 1). In male antennae there was a positively dose-dependent response to *S. litura* sex pheromone gland components, both by those that elicit behavioural responses*,* (9Z,11E)-tetradecadienyl acetate (Z9E11-14:OAc) and (9Z,12E)-tetradecadienyl acetate (Z9E12-14:OAc), and also by the minor components 9Z-tetradecenyl acetate (Z9-14:OAc) and 9E-tetradecenyl acetate (E9-14:OAc) (Figure 1A and B). The sex pheromone isomers 11E-tetradecenyl acetate (E11-14:OAc) and 11Z-tetradecenyl acetate (Z11-14:OAc) elicited significant EAG responses that differed between sexes at the 10^−2^ dosage (v/v) although they are not found in female moths.

### *De novo* transcriptome assembly

A total of 55,288,304 reads of the pooled RNA extract were generated through Illumina sequencing and assembled into 105,971 contigs and then 69,301 unigenes, with a mean length of 603 bp (Additional file 1: Figure S3). More than 39% of all unigenes aligned to sequences in protein databases. The gene ontology (GO) annotation provides information of the gene products, the molecular function, biological process involved and the cellular location. GO annotation makes the transcriptome data more accessible and was used to assess the transcriptome (Additional file 1: Figure S4).

### Analysis of olfactory genes

Twenty six putative OR genes, 21 OBPs, 18 CSPs and 9 IRs were identified for *S. litura* and the mean length of OR, OBP, CSP and IR was 335 aa, 147 aa, 132 aa and 644 aa separately*.* Phylogenetic comparison revealed that 24 (except for OR44 and OR45) of *S. litura* ORs clustered with verified ORs of Lepidoptera (bootstrap value ≥50) (Additional file 1: Figure S5). *Spodoptera litura* ORCO clustered with the ORCO subfamily, SlituOR18 clustered with other lepidopteran OR18 and 5 ORs (OR1, OR11, OR13, OR14, OR23) fell into the pheromone receptors subfamily. Except for the ORCO, ORs of different orders (Lepidoptera, Diptera, Hymenoptera and Hemiptera) were diverged. The twenty-one OBP genes encode 11 OBPs, 4 ABPs, 2 GOBPs, 3 PBPs, and one ABPX. Except for SlituOBP6, all *S. litura* OBP genes were clustered with those of Lepidoptera (bootstrap value ≥50) (Additional file 1: Figure S6). OBPs of different order (Lepidoptera, Diptera, Hymenoptera and Hemiptera) were also diverged. Of the 18 CSP genes of *S. litura* (CSP1-18), 16 (except for CSP2 and CSP3) clustered exclusively (bootstrap value ≥70) with CSPs of Lepidoptera (Additional file 1: Figure S7). One conserved *S. litura* CSP (SlituCSP1) occupied clade with CSPs of *A. mellifera*, *A. pisum* and other Lepidoptera. Nine IRs of *S. litura* were clustered with those of Lepidoptera (bootstrap value ≥70) (Additional file 1: Figure S8). Meanwhile, IR8a and iGLUR6 and their lepidopteran analogues were clusterd with those of *D. melanogaster* with high bootstrap values (≥90).

### Assessment of gene expression in antennae by single-end RNA-Seq

About 6 million clean reads from the single-end RNA-Seq library of each of male and female antennal RNA extracts were generated through Illumina sequencing and, of these, 69.3% and 73.3%, respectively, were uniquely matched with the *de novo* library (Additional file 1: Table S2). For many olfactory genes where RNA-seq reported low gene expression levels, as measured by RPKM values, and where P-values and false discovery rates (FDR) were > 0.05 and/or 0.01 separately, estimates of sex differences in relative expression from RT-qPCR and RNA-Seq differed by a factor of two or more. In these circumstances RT-qPCR gives a more reliable measure and was used in preference. The RT-qPCR is generally considered an efficient, fast, reproducible, reliable and specific for quantifying levels of transcripts [47]. Two reference genes (*GAPDH* and *UCCR*) [48] were chose to perform RT-qPCR according to the MIQE guideline [49] and were used to normalize the data in our study.

### Expression of all olfactory genes in male and female antennae

**Figure 2:**
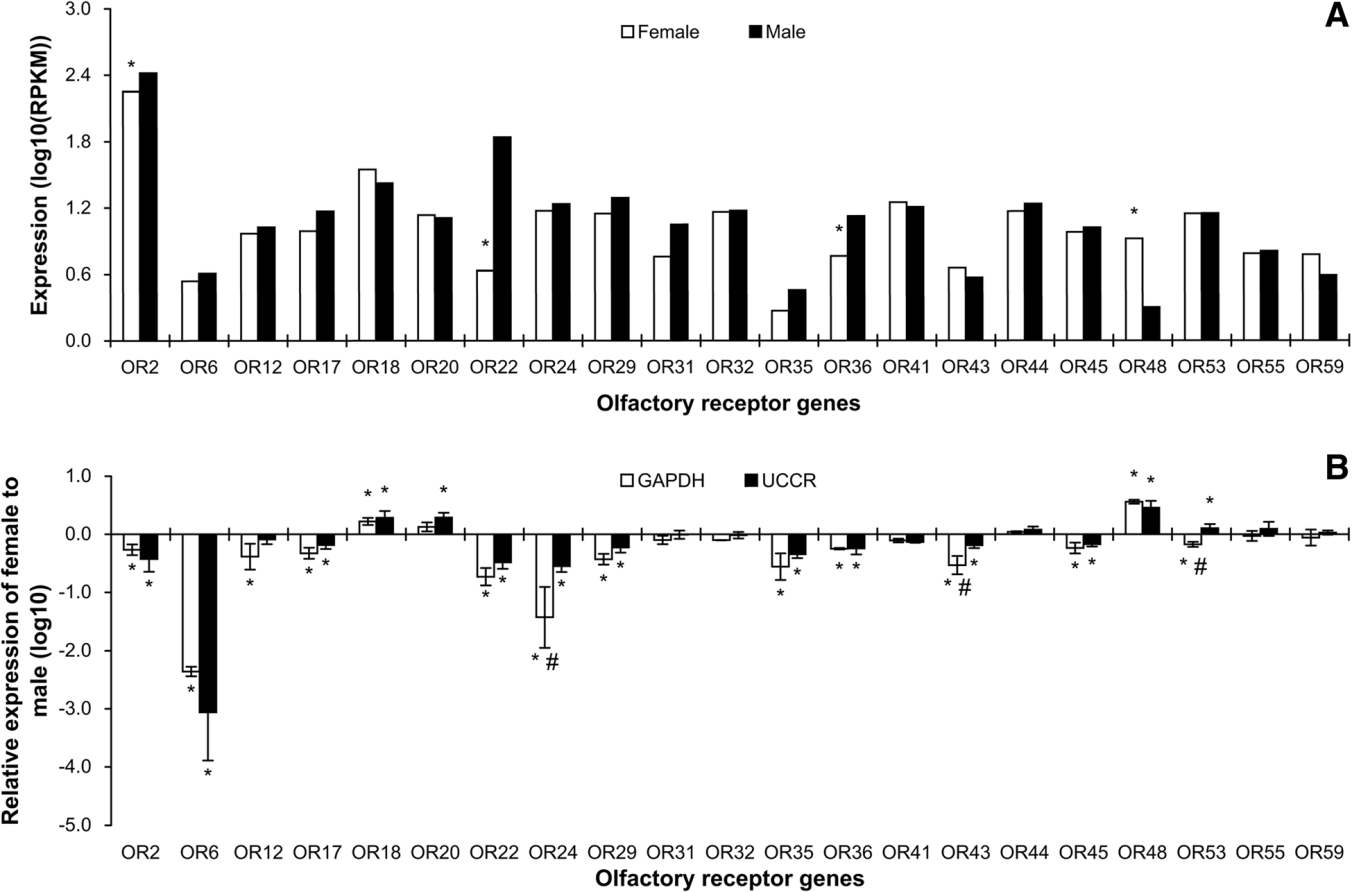
**Expression levels of olfactory genes in male and female** ***S. litura*** **antennae measured in single-end RNA-Seq (A) and RT-qPCR (B).** In single-end RNA-Seq, expression was calculated with log scale of RPKM value. The significant difference between female and male was justified by method of Audic and Claverie (1997) and indicated by symbol “*” (FDR < 0.01 and P < 0.05). In RT-qPCR, gene expression was calculated by the 2^-∆∆Cq^ algorithm with male as control, *GAPDH* and *UCCR* as reference genes. Female gene expression is presented normalized to male antennal expression arbitrarily defined as 1. Error bars signify SD. Significance of difference between male and female responses indicated by *P < 0.05, “#” means the significant difference between *GAPDH* and *UCCR* (P < 0.05), Students *t* test.

**Figure 3:**
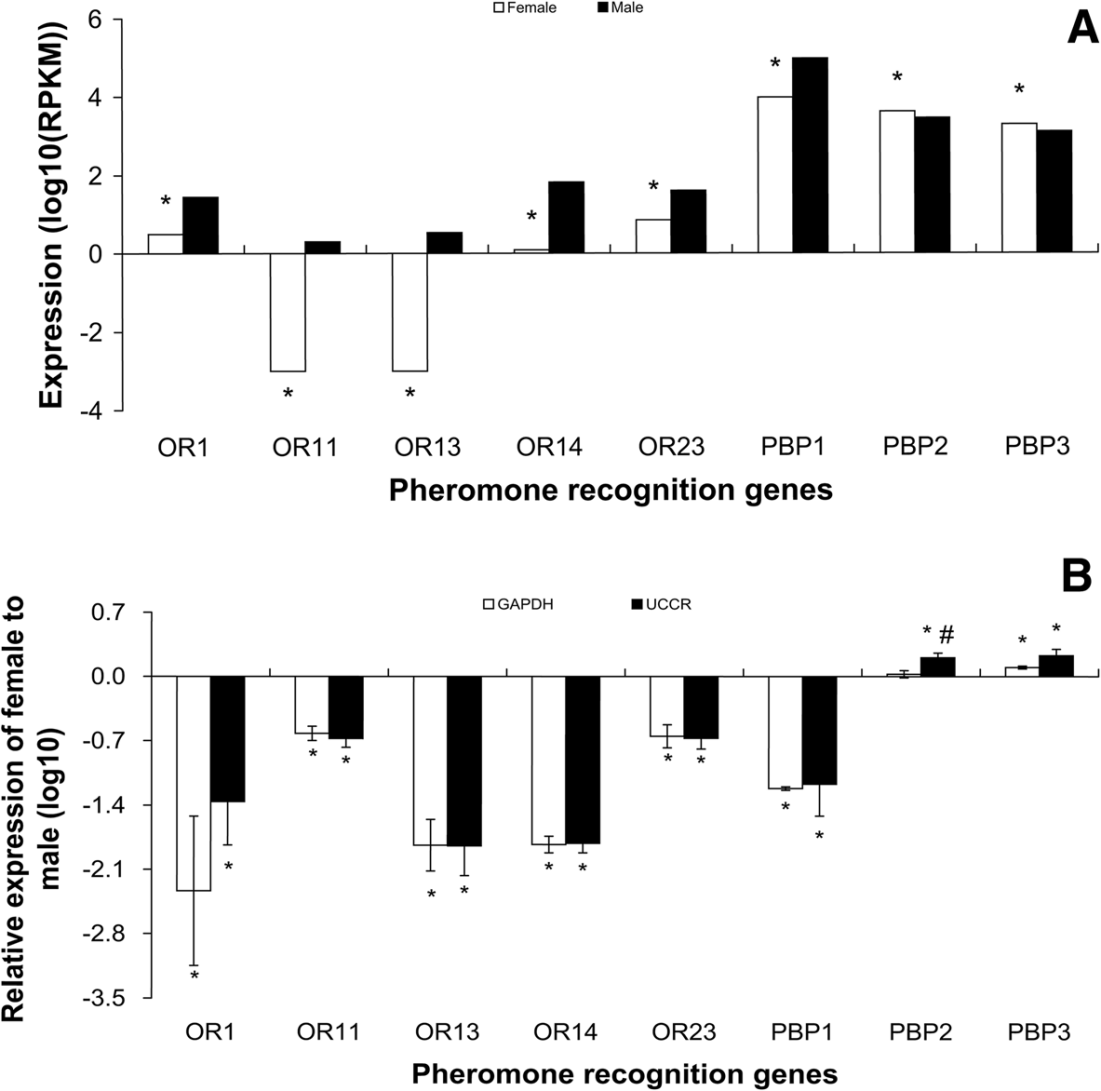
**Expression levels of pheromone recognition genes in male and female** ***S. litura*** **antennae measured in single-end RNA-Seq (A) and RT-qPCR (B).** In single-end RNA-Seq, expression was calculated with log scale of RPKM value. The significant difference between female and male was justified by method of Audic and Claverie (1997) and indicated by symbol “*” (FDR < 0.01 and P < 0.05). In RT-qPCR, gene expression was calculated by the 2^-∆∆Cq^ algorithm with male as control, *GAPDH* and *UCCR* as reference genes. Female gene expression is presented normalized to male antennal expression arbitrarily defined as 1. Error bars signify SD, Significance of difference between male and female responses indicated by *P < 0.05, “#” means the significant difference between *GAPDH* and *UCCR* (P < 0.05), Students *t* test.

**Figure 4:**
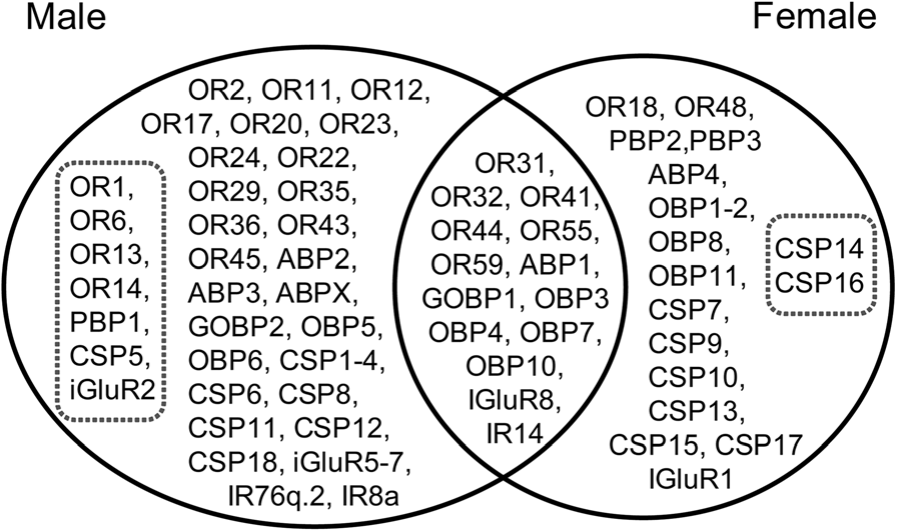
**Summary of differences between male and female** ***S. litura*** **in the levels of antennal expression of candidate olfactory genes based on RT-qPCR.** Genes in the overlapping intersect between sexes show no significant difference between male and female. Genes outside the intersect between sexes show significant difference between sexes. Those in the dash-outlined area show more than tenfold greater expression in male or female antenna and can be considered sex-specific. *OR53* and *OBP9* were not included because of contradictory results of two reference genes or RT-qPCR failed.

Expression levels of putative OR genes were low. The RPKM values for all ORs were less than 70, except for *ORCO* which had values of 179 and 262 for female and male antennae, respectively (Figure 2A). Four of the sex differences of ORs in expression shown by RNA-Seq were confirmed by RT-qPCR (Figure 2). Most of sex differences of ORs in expression shown by RNA-Seq were confirmed by RT-qPCR (Figure 2). Of 21 recognized general ORs, RT-qPCR showed 2 to be significantly more expressed in female antennae, relative to the standard *GAPDH* and *UCCR* gene, and 170 were significantly more expressed in male antennae (Figure 2B). Like other ORs, expression levels of candidate pheromone receptors measured in the RNA-Seq analysis were low (≤80 RPKM) (Figure 3A). However, expression of *OR23* and *OR14* in males was markedly higher than for the other 3 candidate pheromone receptors (Figure 3A) and expression of *OR14* was amongst the most male-biased. RT-qPCR confirmed the markedly higher relative expression of all candidate pheromone receptors in male antennae (Figure 3B). Usually the difference of general ORs between the sexes was less than four fold. However, *OR6* was predominantly expressed in the male compared to the female antennae (Figure 4).

**Figure 5:**
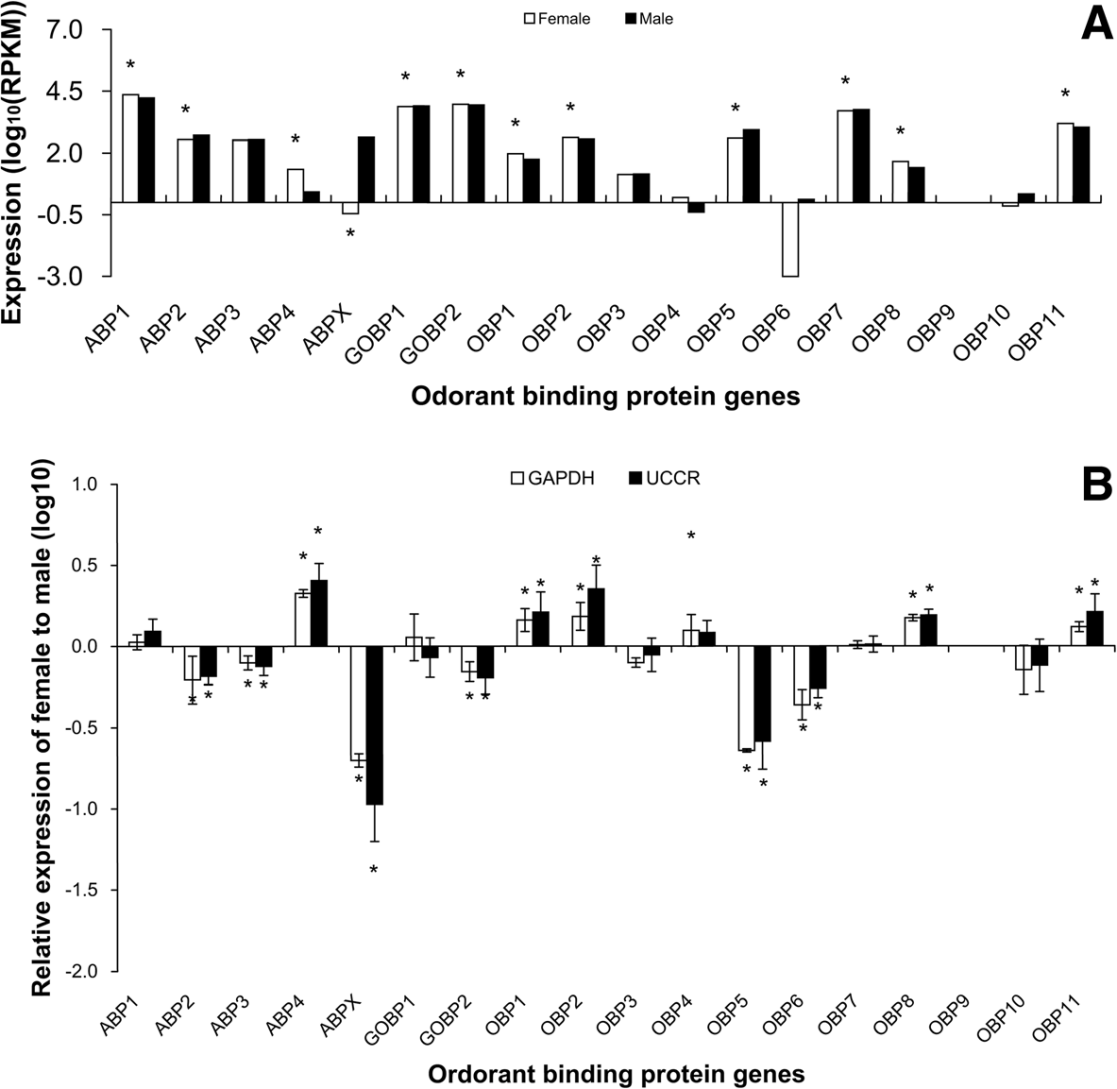
**Expression levels of candidate OBPs in male and female** ***S. litura*** **antennae measured in single-end RNA-Seq (A) and RT-qPCR (B).** In single-end RNA-Seq, expression was calculated with log scale of RPKM value. The significant difference between female and male was justified by method of Audic and Claverie (1997) and indicated by symbol “*” (FDR < 0.01 and P < 0.05). In RT-qPCR, gene expression was calculated by the 2^-∆∆Cq^ algorithm with male as control, *GAPDH* and *UCCR* as reference genes. Female gene expression is presented normalized to male antennal expression arbitrarily defined as 1. Error bars signify SD, significance of difference between male and female responses indicated by *P < 0.05.

**Figure 6:**
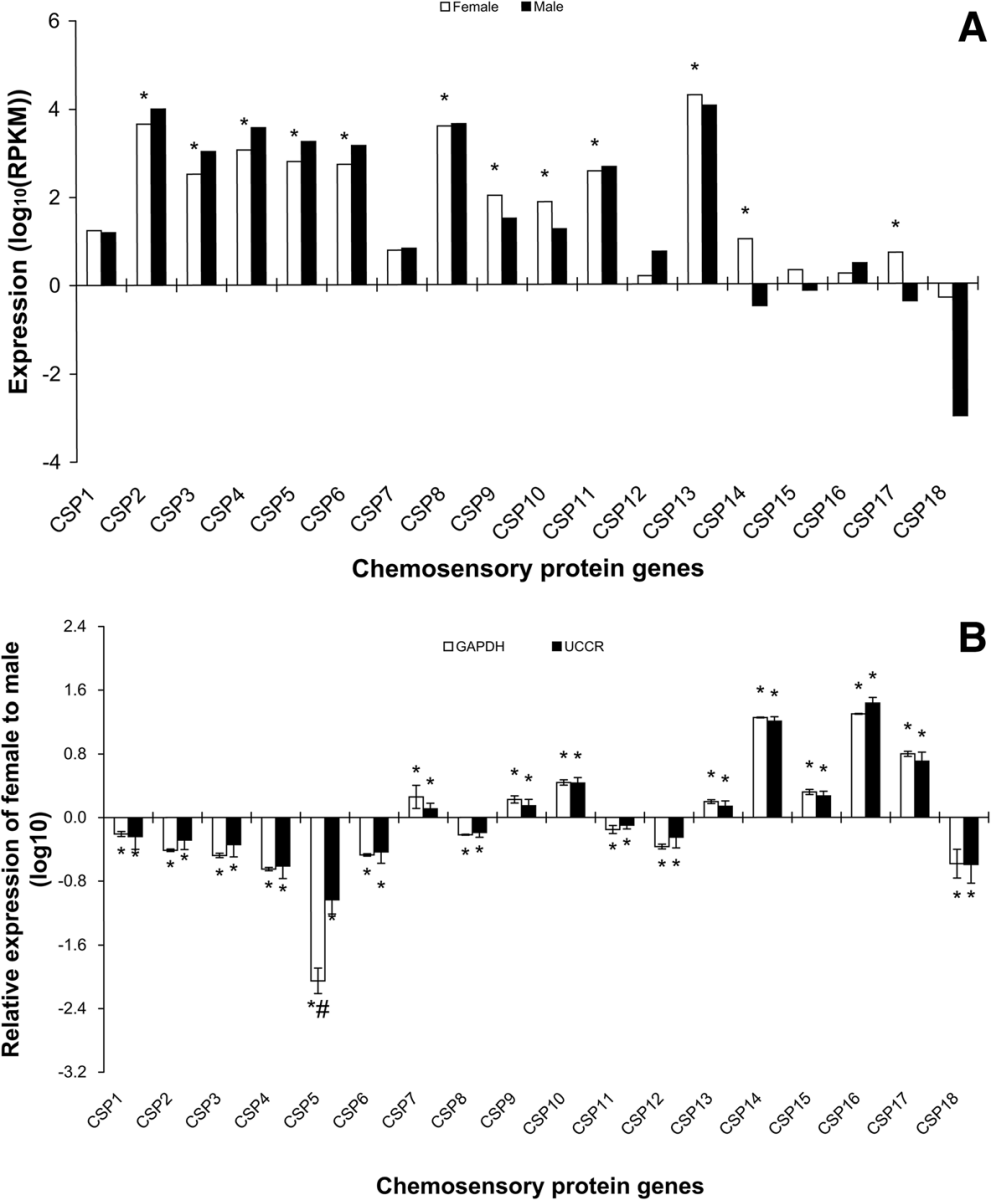
**Expression levels of candidate CSPs in male and female** ***S. litura*** **antennae measured in single-end RNA-Seq (A) and RT-qPCR (B).** In single-end RNA-seq, expression was calculated with log scale of RPKM value. The significant difference between female and male was justified by method of Audic and Claverie (1997) and indicated by symbol “*” (FDR < 0.01 and P < 0.05). In RT-qPCR, gene expression was calculated by the 2^-∆∆Cq^ algorithm with male as control, *GAPDH* and *UCCR* as reference genes. Female gene expression is presented normalized to male antennal expression arbitrarily defined as 1. Error bars signify SD. Significance of difference between male and female responses indicated by * P < 0.05, “#” means the significant difference between *GAPDH* and *UCCR* (P < 0.05), Students *t* test.

**Figure 7:**
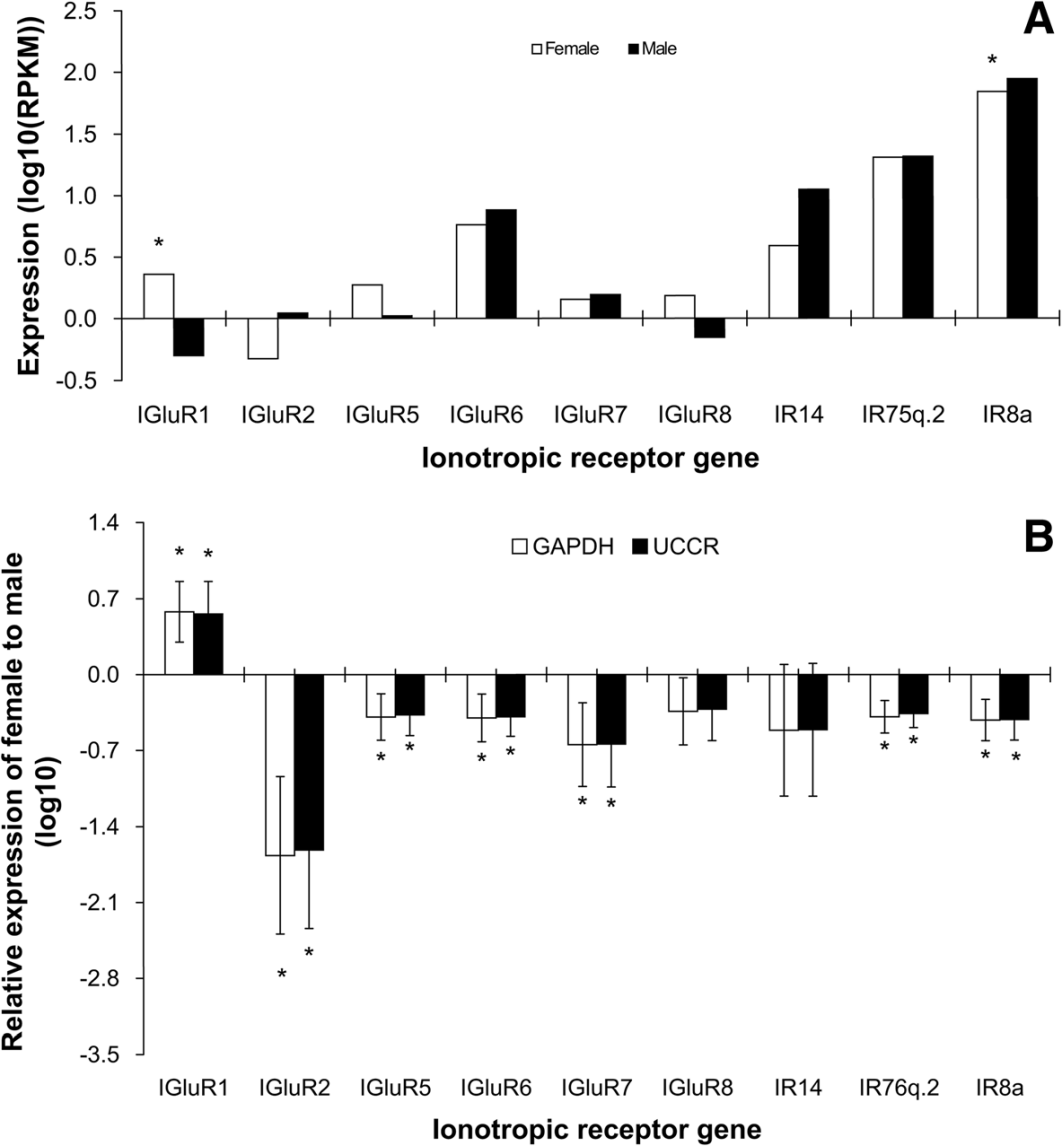
**Expression levels of candidate IRs in male and female** ***S. litura*** **antennae measured in single-end RNA-seqence (A) and RT-qPCR (B).** In single-end RNA-seqence, vertical axis means the log scale of RPKM value to 10. The significant difference between female and male was justified by method of Audic and Claverie (1997) and indicated by symbol “*” (FDR < 0.01 and P < 0.05). In RT-qPCR, vertical axis means log scale of female gene expression to 10. Female gene expression was calculated by the 2^-∆∆Cq^ algorithm with male as control and *GAPDH* and *UCCR* as reference genes. The significant difference was ascertained by Students *t* test. Symbol “*” means the significant difference between female and male (P < 0.05). Error bars signify SD”.

By contrast with candidate pheromone receptors, the expression levels of candidate pheromone-binding protein (PBP) genes in antennae were very high, the male expression of PBP1 being the highest expression of all olfactory genes at 94161 RPKM (Figure 3A). The sex-bias expression varied greatly between the three PBPs, being strongly male-biased for PBP1 and female biased for PBP3 (Figure 3A). The levels of expression of other odorant binding proteins in the antennae were extremely variable, with RPKM values ranging from less than 50 to over 20,000 (Figure 5A). Five OBPs were shown to be more highly expressed in female antennae and 3 OBPs to be more highly expressed in male antennae in single-end RNA-Seq and RT-qPCR (Figure 5). However, the total number of male-biased OBPs were the same as that of female (Figure 4). The range of expression levels of CSP genes in the antennae was as extreme as for OBPs, RPKM values ranging from less than 10 to almost 20,000 (Figure 6A). Twelve CSP genes showed sex differences in their levels of antennal expression, 5 being more expressed in females and 7 more expressed in males and sometimes these differences were marked (Figure 6). The expression levels of IR genes were as low as those of ORs and the largest RPKM value was about 1000 (Figure 7A). Of which, IGluR1 was female biased and IR8a was male biased (Figure 7). Only the expression levels of 3 ORs, *PBP2* and *CSP5* showed significant difference by using *GAPDH* and *UCCR* as reference genes.

## Discussion

*Spodotera litura* is a polyphagous and widely distributed agricultural pest that shows antennal responses to a broad range of chemicals. Until now the genes encoding its olfaction-related proteins have been little known. In this paper we have characterized the olfactory genes of *S. litura* and their antennal expression using transcriptome analysis, single-end RNA-Seq, and RT-qPCR. We found 74 olfactory genes in the antennae, including 26 ORs, 21 OBPs, 18 CSPs and 9 IRs. Antenal transcriptome was reported in the moths *M. sexta* (48 ORs, 18 OBPs, 21 CSPs and 4 IRs) [12] and *B. mori* (71 ORs, 20 OBPs, 16 CSPs and 31 IRs) [11,25]. In the congeneric species, *S. littoralis*, 46 ORs, 35 OBPs, 21 CSPs and 5 IRs have recently been found in antennae transcriptome [18,50,51]. The surprisingly large difference in the number of olfactory genes obtained for the two *Spodoptera* species may not be due to the database size but the methodological differences of olfactory genes analysis in the two studies. The *S. littoralis* study obtained 77,643 contigs with a median size of 653 bp [18,51]. In our study, it was 105,971 contigs with a median size of 645 bp, which assembled into 69,301 unigenes with a median size of 974 bp. We adopted a more strict criteria to select candidate olfactory genes, a 50% average ORF length (1200 bp) cutoff as a putative gene. The mean length of OR, OBP, CSP and IR genes was 335 aa, 147 aa, 132 aa and 644 aa respectivelyin our study, which was close to the full length of each category.

Phylogenetic analysis showed that some *S. litura* olfactory genes clustered not only with those of other Lepidoptera but also with those of *A. mellifera*, *D. melanogaster* and *A. pisum* indicating a certain degree of conservation typical of olfactory gene families. The extent of gene conservation is likely to reflect function. For example, the role of OBPs is to assist hydrophobic odorant molecules to cross the aqueous barrier at the pore of the sensillum to reach and bind to ORs on the dendrite of the olfactory neuron [13]. OBPs are relatively generalist, binding more than one molecule [52], and so they are likely to be conserved across larger taxonomic groupings. A recent study by McKenzie *et al*. showed that both OBPs and CSPs expressed specifically in antennae of the *Cerapachys biroi* possibly serve the olfactory functions [53]. By contrast, ORs often specifically respond to one particular odorant and those that play a key roles in the autecology of a species are likely to be less conserved across taxa, particularly if they contribute to the reproductive isolation of species as do sex pheromone receptors [54]. ORs that respond to odors common across habitats such as certain green leaf volatiles may be more conserved.

The phylogenetic distribution of *S. litura* ORs was consistent with other Lepidoptera and the relatively conserved nature of much of the OR gene family. Five *S. litura* ORs clustered together with a conserved OR subfamily of moth sex pheromone receptors [55] on an exclusively lepidopteran branch of the phylogenetic tree. *S. litura* ORCO clustered with the conserved ORCOs, co-receptors for odor- and pheromone-specific ORs [56]. The noctuid moths have another conserved subfamily, OR18 [54]. The OBP family comprises OBPs, ABPs and three subfamilies conserved within Lepidoptera PBP, GOBP, and ABPX [30,57]. *S. litura* OBPs fell both within and outside these subfamilies and clustered with other Lepidoptera. One of *S. litura’s* 18 CSPs clustered with a CSP subfamily which is highly conserved across insects and the remainder clustered with lepidopteran CSPs.

In *S. litura* we found the number of candidate pheromone receptor genes (five) close to there are components of the sex pheromone gland (Z9E11-14:OAc, Z9E12-14:OAc, Z9-14:OAc, and E9-14:OAc) that are active in EAG [41]. Multiple sex pheromone receptors (e.g. [35,52,58] and multi-component sex pheromones (e.g. [59-61]) are found in other moths and the excess of pheromone receptor genes is not unique to *S. litura* [59]. Sex pheromone receptors are very specific and so one might expect their number to equal the number of sex pheromone components. It is possible that multiple pheromone receptors may be involved in identifying each component. Using heterologous expression in *Xenopus* oocytes, both *SexiOR13* and *SexiOR16* of *S. exigua* respond to Z9E12-14:OH [62]. Alternatively, during evolution some components may have been lost from the pheromone gland before the corresponding receptor was lost from the antenna [63]. This might explain the EAG response in *S. litura* to Z11-14:OAc and E11-14:OAc, compounds related to known components of the sex pheromone gland but not themselves present. Retention of the ability to recognise pheromone components that no longer signify conspecific females may assist in the maintenance of reproductive isolation of species.

There were dramatic differences in levels of expression of the diverse olfactory genes in *S. litura* and some of these can readily be related to function. The 15 most highly expressed genes (RPKM larger than 1000) were all binding proteins (OBPs, CSPs and PBPs). By contrast, most ORs and IRs had RPKM values less than 50. OBPs are usually highly expressed and solubilizing in sensillar lymph. OBPs bind with multiple odorants, and are fewer in number. The most highly-expressed binding protein was a pheromone-binding protein, *PBP1*, and candidate pheromone receptors were among the most highly-expressed ORs. This reflects the value of being able to detect very small amounts of pheromone [59]. The most highly expressed OR was *ORCO*, consistent with evidence from phylogenetic studies [45].

We found many sex differences in expression levels of olfactory genes in *S. litura*, some of them extreme. Many olfactory responses are common to both sexes, such as those to many host plant volatiles, and this is reflected in the third of OR genes that we found equally expressed in both sexes. Others, particularly those involved in mating or oviposition behaviours, are likely to be sex-specific. Using RT-qPCR, we found a strong bias towards males in the number of OR genes with sex-specific expression, 15 showing significantly more expression in males and only 2 being more expressed in females. Figure 4 summarises sex differences in olfactory gene expression. The majority of genes fall into the area where sex differences in expression are less than ten-fold but 4 ORs were at least ten-fold as much expressed in males. This imbalance may be associated with male responsiveness to female sex pheromone, yet the number of ORs that show male-biased expression is well in excess of the 5 putative pheromone receptors we identified.

Our EAG studies support the conclusion that sex-biased expression of ORs is related to function and, at least in part, to the male response to sex pheromone. The EAG measures the sum of neuronal activity in the antennae reflecting the integrated response of olfactory receptors to a volatile, other related genes might also be involved in that responses. Of the 58 volatiles tested by EAG, 19 evoked a significantly sex-biased response and in each case greater electrical activity was recorded in male antenna. Of these, the strongest EAG responses were to the two behaviorally-active sex pheromone components, Z9E11-14:OAc, Z9E12-14:OAc. The two highly-expressed receptors found here, *OR14* and *OR23*, showed strongly male-biased (Figure 3B) and it is possible that they are the receptors for Z9E11-14:OAc and Z9E12-14:OAc. Further studies are to be performed to confirm these two receptors are the receptors respond to pheromone components. Many ORs that were not candidate pheromone receptors also showed male-biased expression (Figure 2). An increase in the expression of genes that don’t have a sex-specific function might in part be a by-product of the elaboration of the male antenna that enables it to carry abundant pheromone-sensitive sensilla. The sex-biased expression of CSPs (10 male-biased, 8 female-biased) indicate that CSPs play differential roles in the male and female moths. There was no sex bias in the number of OBP genes that showed sex-specific expression, probably reflecting their less specific role as binding proteins. However, the most highly expressed OBP was a pheromone binding protein, *PBP1*, which was ten times more expressed in males, suggesting that it is involved in detection of the sex pheromone. Moreover, our data obtained from the field trials showed that some of *S. litura* pheromone isomers play a sygnergistic or antagonistic role when mixed into the sex pheromone blend (Du *et al.*, unpublished result). We infers that the ORs or OBPs could be related to recognition of those pheromone isomers and the interspecific communications.

## Conclusions

In summary, we have identified the 26 olfactory receptor genes, 21 odorant-binding protein genes, 18 chemosensory protein genes and 9 ionotropic receptor genes that are key to understanding the molecular basis of olfactory responses to sex pheromones and plant volatiles in *S. litura.* Transcriptome and expression profiling analyses revealed variation in gene expression, often sex-biased, that was reflected in the strength of antennal responses and may lead to the functional identification of genes. Our results pave the way for future elucidation of the molecular basis of olfactory and mating behaviors of this moth, and the development of new biorational pheromone technologies that target particular genes, proteins and behaviors for pest monitoring and control.

## Methods

### Insects

*Spodoptera litura* (Lepidotera, Noctuidae) pupae were purchased from the Institute of Zoology, Chinese Academy of Science, and lab reared. For details see Additional file 1: *Materials and Methods.*

### EAG recording

Recordings of whole-antenna electrical activity in response to volatile stimuli were made according to standard techniques [64,65]. Antennae were challenged with 58 volatile chemicals presented singly and selected from flowers, host or non-host plants, and the sex pheromone components of *S. litura* and their isomers, some of which are sex pheromones of other moths (Additional file 1: Table S1). Each chemical was dissolved in paraffin oil and tested at two concentrations, 10^−4^ v/v and 10^−2^ v/v. A 10-μl aliquot of paraffin oil on the filter paper was used as the control. The responses of antennae from ten male and ten female moths were tested for each treatment. For further details see Additional file 1: *Materials and Methods*.

### Extraction of total RNA from tissues

To obtain complete gene expression information in the transcriptome analysis, RNA was extracted separately from different developmental stages and sexes and then pooled. Separate RNA extracts were made of the antennae of each sex for expression profiling analysis and for RT-qPCR. Total RNA was extracted using RNAiso Plus (Takara, China). For further details see Additional file 1: *Materials and Methods*.

### Transcriptome de novo analysis

The cDNA libraries for transcriptome analysis were prepared using TruSeq SBS Kit v3-HS (Illumina, America) following manufacturer’s recommendations. The libraries were sequenced using Illumina HiSeq™ 2000 (Illumina, America) with 90 bp read length of reads-paired end. Dirty reads containing adapters and unknown or low quality bases were discarded from raw reads to obtain clean reads for analysis. Transcriptome *de novo* assembly was carried out with the short reads assembling program, Trinity [66]. Blastx alignment (E value < 0.00001) between unigenes and protein databases (NCBI non-redundant protein database, Swiss-Prot, KEGG and COG) was successively performed. When a unigene could not be aligned to any of the databases, ESTScan software was used to decide its sequence direction and the predicted coding region [67]. Gene ontology (GO) annotations of the unigenes were determined using Blast2go (https://www.blast2go.com/) [68]. WEGO software was used for GO functional classification for all unigenes and to understand the distribution of gene function at the macro level [69]. The raw sequence of the transcriptome has been deposited in the National Center for Biotechnology Information (NCBI) (accession number: PRJNA273435; http://www.ncbi.nlm.nih.gov/bioproject/273435). For further details see Additional file 1: *Materials and Methods*.

### Olfactory gene analysis

The candidate olfactory gene was obtained from GO annotation. In addition, a 50% ORF length cutoff was used for considering a putative gene to prevent a gene from being counted twice. Amino acid sequence alignment were performed using clustalx [70]. For the phylogenetic analysis, amino acid sequences of ORs, CSPs and OBPs of *D. melanogaster* [22,38], *Apis mellifera* [71], *Acyrthosiphon pisum* [72], *Bombyx mor* [8,11], *Manduca sexta* [73], *Spodoptera littoralis* [18,51] and *Heliothis virescens* [14,32], and IRs of *D. melanogaster* [36], *Bombyx mor* [25], *Manduca sexta* [73], *Spodoptera littoralis* [18,51] and *Helicoverpa armigera* [37] were used. Phylogenetic analyses were conducted with maximum likelihood method of MEGA 6.0 based on Jones-Taylor-Thornton (JTT) substitution model, partial deletion gaps with 95% site coverage cutoff and Nearest Neighbour Interchanges (NNI) heuristic search [74]. Node support of phylogenetic tree was assessed using the bootstrap method with 100 bootstrap replicates.

### Profiling analysis of antennal gene expression using single-end RNA-Seq library

Clean reads were mapped to *de novo* library sequences using SOAP2 [75]. Sequence saturation analysis was used to measure the sequencing data. The distribution of reads locating on reference genes was used to evaluate the randomness of fragmentations [76]. The gene expression level was calculated using the RPKM method [77] to take account of differing gene lengths. The raw sequence has been deposited in NCBI as above. For further details see Additional file 1: *Materials and Methods*.

### RT-qPCR analysis of olfactory gene expression in antennae

RT-qPCR was performed on total RNA of male and of female antennae to validate between-sex comparisons of gene expression made using single-end RNA-Seq data and extend them to all candidate olfactory genes, including those with lower expression levels. The PCR primers used are listed in Additional file 1: Table S3. Six or more replicates were made. The data were analyzed using SPSS 17.0. For further details see Additional file 1: *Materials and Methods*.

### Statistical analysis

Data analysis was conducted using SAS 9.2. Significance of the difference between means was determined by Student’s *t*-test.

## Additional file

Additional file 1:
**Supporting information.**

## Abbreviations

E9-14:OAc: 9E-tetradecenyl acetate
Z9-14:OAc: 9Z-tetradecenyl acetate
Z9E11-14:OAc: (9Z,11E)-tetradecadienyl acetate
Z9E12-14:OAc: (9Z,12E)-tetradecadienyl acetate
E11-14:OAc: 11E-tetradecenyl acetate
Z11-14:OAc: 11Z-tetradecenyl acetate
ABPs: Antennal-binding proteins
CSPs: Chemosensory proteins
EAG: Electroantennogram
FDR: False discovery rates
GO: Gene ontology
GOBPs: General odorant-binding proteins
IRs: Ionotropic receptors
ORs: Olfactory receptors
ORCO: Olfactory receptor coreceptors
OBPs: Odorant-binding proteins
PBPs: Pheromone-binding proteins
RT-qPCR: Reverse transcription–quantitative real-time PCR
RPKM: Reads Per Kb per Million reads
