# Supplementary material for: Transcriptome and expression profiling analysis link patterns of gene expression to antennal responses in Spodoptera litura
Source: BMC Genomics. 2015 Apr 7;16(1):269. doi: 10.1186/s12864-015-1375-x (PMC4502548; doi:10.1186/s12864-015-1375-x)
Supplement: Additional file 1: — Supporting information. [file 12864_2015_1375_MOESM1_ESM.doc]

**Supporting Information**

**Materials and methods**

**Insects.** *Spodoptera litura* (Lepidotera, Noctuidae) pupae were purchased from the Institute of Zoology, Chinese Academy of Science, and lab reared at 25±1°C and 75±5% relative humidity with a 14 h photoperiod. Males and females were maintained separately in ventilated wooden cages (35cm×35cm×50cm). Emerging adult moths were fed with 10% glucose water soaked into cotton. Five pairs of 2-day-old adults were mated in a cage and the eggs, which were deposited on paper, were collected. Hatching larvae were fed with artificial diet prepared according to a published recipe based on chickpea flour, kidney bean flour, yeast powder, wheat germ, and water with added vitamins and minerals .

**Electroantennogram (EAG) recording**. Antennae for EAG recordings were amputated from the head at their base. A reference electrode was inserted into the base of the antenna, and a recording electrode was in contact with the tip of the antenna. Each electrode consisted of a 0.25 mm inside-diameter glass capillary tube filled with Kaissling electrophysiological solution, containing 354 mM glucose, 1 mM calcium chloride, 20 mM potassium dihydrophosphate, 6.4 mM potassium chloride, 12 mM magnesium chloride, 12 mM sodium chloride, with potassium hydroxide to adjust the pH to 6.5 . A silver-silver chloride wire (0.4 mm diameter) inside each glass capillary was connected to the pre-amplifier of a Syntech EAG recording system. From there, signals were transmitted to an oscilloscope for monitoring and through a Syntech signal acquisition controller, IDAC-2, to a computer for recording and analysis using Syntech EAG 2000 software. Stimuli were presented by introducing odoriferous air into a continuous air stream (1200 ml/min) cleaned by activated charcoal, humidified by passage through water, and directed at the antenna through a Pasteur pipette. Odours were introduced in air passed through a glass dropper containing a 6cm × 0.5cm strip of filter paper impregnated with 10μl of a solution of the test compound in paraffin oil. For each stimulus, a 0.1 s air puff of odoriferous air (air flow 40 ml/min) was delivered into a hole in the Pasteur pipette carrying the clean air stream. To avoid sensory adaptation, the interval between two stimuli was at least 30 s.

**Extraction of total RNA from tissues.** Total RNA was extracted from the following tissues that had been stored in liquid nitrogen for this purpose: nearly 400 eggs, five 4^th^ instar larvae, 40 heads of 4^th^ instar larvae, five 3-day-old pupae of each sex, five 2-day-old adults of each sex, and the antennae of 50 2-day-old adults of each sex. Separate RNA extracts were made of the antennae of 1500 2-day-old adults of each sex for expression profiling analysis of the antennae, and of a further 200 of each sex for quantitative real-time RT-PCR of the antennae. Total RNA was extracted from each sample using RNAiso Plus (Takara, China) and extracts were treated with DNase I (Takara, China) to remove any DNA, in each case following the manufacturer’s protocols. The concentration of total RNA in each extract (not less than 200 ng/µl per tissue) was determined by spectrophotometer (U-0080D, Hitachi).

**RNA-seq library preparation.** The cDNA library for transcriptome analysis were prepared using TruSeq SBS Kit v3-HS (Illumina, America) following manufacturer’s recommendations. Briefly, Oligo (dT) magnetic beads were used to isolate poly(A) mRNA from total RNA collected. Fragmentation buffer was added to break mRNA into short fragments (200-700nt). Random hexamer primer was used to synthesize the first-strand cDNAs with short RNA fragments as templates. The second-strand cDNAs were synthesized using buffer, dNTPs, RNase H and DNA polymerase I. The resulting short double-stranded cDNAs were purified and eluted with elution buffer for end preparation and to add poly(A). The short cDNAs were then linked to sequencing adapters. The required fragments were purified by agarose gel electrophoresis and enriched by PCR amplification.

**Paired-end RNA-seq and transcriptome de novo assembly.** The library was sequenced using Illumina HiSeq™ 2000 with 90 bp read length of paired end. Image data output from sequencing machine is transformed by base calling into raw reads. Clean reads were obtained when reads with adaptors, reads with unknown nucleotides larger than 5% and reads with low quality (more than 50% bases with quality value ≤ 5 in a read) were removed from raw reads. Transcriptome *de novo* assembly was carried out with the short reads assembling program, Trinity . Briefly, overlap reads were first combined to form contigs and then the reads were mapped back to contigs. With paired-end reads, we were able to detect contigs from the same transcript as well as the distances between these contigs. Finally, the contigs were connected to form unigenes that could not be extended at either end.

**Gene annotation.** Blastx alignment (E value < 0.00001) between unigenes and protein databases (nr, Swiss-Prot, KEGG and COG) was successively performed, and the best aligning results were used to decide sequence direction and coding region of the unigenes. If unigenes aligned to a higher priority database (in this order of priority: nr, Swiss-Prot, KEGG and COG), results from lower priority databases were not used. When a unigene could not be aligned to any of the databases, ESTScan software was used to decide its sequence direction and the predicted coding region . Gene ontology (GO) annotations of the unigenes were determined using Blast2go (<http://www.blast2go.org/>) . After getting GO annotation for every unigene, we used WEGO software to do GO functional classification for all unigenes and to understand the distribution of gene functions of the species at the macro level .

S**ingle-end RNA-seq of library from antennae and reads assembly.** 49 bp read length of single end was sequenced using Illumina HiSeq™ 2000. Dirty reads were discarded from raw reads, and the clean reads obtained were mapped to *de novo* library sequences using SOAP2 . Mismatches with no more than two bases were allowed in the alignment. All the reads that mapped to multiple genes in reference sequences were filtered out, and the remaining reads were designated as unambiguous reads. Sequence saturation analysis was used to measure the sequencing data. The distribution of reads locating on reference genes was used to evaluate the randomness of fragmentations .

**Profiling analysis of gene expression in antennae**. The number of expressed reads for a gene is influenced by the gene length. Therefore the gene expression level was calculated using the RPKM (Reads Per Kb per Million reads) method in which the number of mapped reads per million reads for a gene is divided by the length of that gene. If there were multiple transcript variants for a gene, the longest one was used to calculate its expression level. The identification of differentially expressed genes between two samples was referred to the method of Audic and Claverie and P-value corresponded to differential gene expression test . P-value is the probability of gene A expressed equally between male and female antennae. P-value can be calculated with: P (y|x)=(N2/N1)^y^(x+y)!/[x!y!(1+N2/N1)^(x+y+1)^]. N1 and N2 represented the total clean tag numbers of male and female antennae seperately, x and y represented the tag numbers of gene A in the male and female antennae seperately. The false discovery rate (FDR) is a method to determine the threshold of P-value in multiple tests . P-value and FDR value were calculated to determine the differentially expressed genes in the study with "FDR < 0.01 and P<0.05".

**RT-qPCR of olfactory gene expression in antennae.** Reverse transcription–quantitative real-time PCR (RT-qPCR) was performed on total RNA of male and of female antennae. Single-stranded cDNAs were synthesized from 2 μg of total RNA with PrimeScript Reverse Transcriptase (TaKaRa, Japan) using the protocol supplied with the kit. RT-qPCR was performed with SsoFast™ EvaGreen® Supermix (Bio-Rad), following the manufacturer’s protocols, in a CFX-96™ PCR Detection System (Bio-Rad). The PCR primers used are listed in Table S3. Glyceraldehyde 3-phosphate dehydrogenase (*GAPDH*) and ubiquinol-cytochrome c reductase (*UCCR*) were used as reference genes . The difference in gene expression between male and female antennae was measured by the 2^-∆∆Cq^ algorithm with male antennae as the control. RNA was extractd from three samples of antennae and there were two or more replicates RT-qPCR for every sample. The data were analyzed using SPSS 17.0. Significance of difference between data was conducted by independent-samples T test in the level of 0.05.

**Figure legends**

**Fig. S1** A world map of *S. litura* distribution based on CABI web information (http://www.cabi.org/isc/datasheet/44520#toDistributionMaps)

**Fig. S2** Electroantennogram responses recorded from male and female *S. litura* antennae elicited by: A and B sex pheromones isomers; C and D floral scents and plant volatiles. A and C stimulating dosage 10^-4^ v/v, B and D stimulating dosage 10^-2^ v/v, see Methods and materials for details. Error bars signify SEM.

**Fig. S3**  Size distribution of all 69,301 unigenes assembled from the pooled *S. litura* RNA extract

**Fig.** S**4**  Gene ontology (GO) analyses of transcriptome data from pooled sample of RNA from all life stages and both sexes. GO analysis of sequences corresponding to 8,632 contigs, as predicted for their involvement in biological processes (A) and molecular functions (B), is shown. Data are presented as level 2 GO categorization for biological process and molecular function. Classified gene objects are depicted as percentages of the total number of gene objects with GO assignments.

**Fig. S5** Aligned putative OR gene sequences of *Spodoptera litura*, *Drosophila melanogaster* (black lines), *Apis mellifera* (green lines), *Acyrthosiphon pisum* (yellow lines) and Lepidoptera (red lines). A triangle indicates a compressed clade. Subfamily ORCO, subfamily pheromone receptor and clade OR18 were marked by dot-[trapezium](http://www.iciba.com/trapezium). Number in phylogenetic tree means bootstrap values and bootstrap values lower than 50% are hidden. Bmor: *Bombyx* *mor*, Msex: *Manduca sexta*, Slit: *Spodoptera littoralis*, Hvir: *Heliothis virescens*, Slitu: *S. litura*.

**Fig. S6** Aligned putative OBP gene sequences of *Spodoptera litura*, *Drosophila melanogaster* (black lines), *Apis mellifera* (green lines), *Acyrthosiphon pisum* (yellow lines) and Lepidoptera (red lines). A triangle indicates a compressed clade. PBP-GOBP family was marked by dot-[trapezium](http://www.iciba.com/trapezium). Number in phylogenetic tree means bootstrap values and bootstrap are hidden when they are lower than 50%. Bmor: *Bombyx* *mor*, Msex: *Manduca sexta*, Slit: *Spodoptera littoralis*, Hvir: *Heliothis virescens*, Slitu: *S. litura*.

**Fig. S7** Aligned putative CSP gene sequences of *Spodoptera litura*, *Drosophila melanogaster* (black lines), *Apis mellifera* (green lines), *Acyrthosiphon pisum* (yellow lines) and Lepidoptera (red lines). A conserved subfamily (subfamily I) was marked by dot-trapezium. Number in phylogenetic tree means bootstrap values and bootstrap values are hidden when they are lower than 50%. Bmor: *Bombyx* *mor*, Msex: *Manduca sexta*, Slit: *Spodoptera littoralis*, Hvir: *Heliothis virescens*, Slitu: *S. litura*.

**Fig. S8** Alignment of *Spodoptera litura* IR genes, *Drosophila melanogaster* (black lines) and Lepidoptera (red lines). Number in phylogenetic tree means bootstrap values and bootstrap values are hidden when they are lower than 60%. Bmor: *Bombyx* *mor*, Dmel: *Drosophila melanogaster*, Harm: *Helicoverpa armigera*, Msex: *Manduca sexta*, Slit: *Spodoptera littoralis*, Slitu: *S. litura*.

**Table legends**

**Table S1** List of all compounds tested in EAG

**Table S2** Single-end RNA-seq: reads and mapping data

**Table S3** Primers used for qPCR.


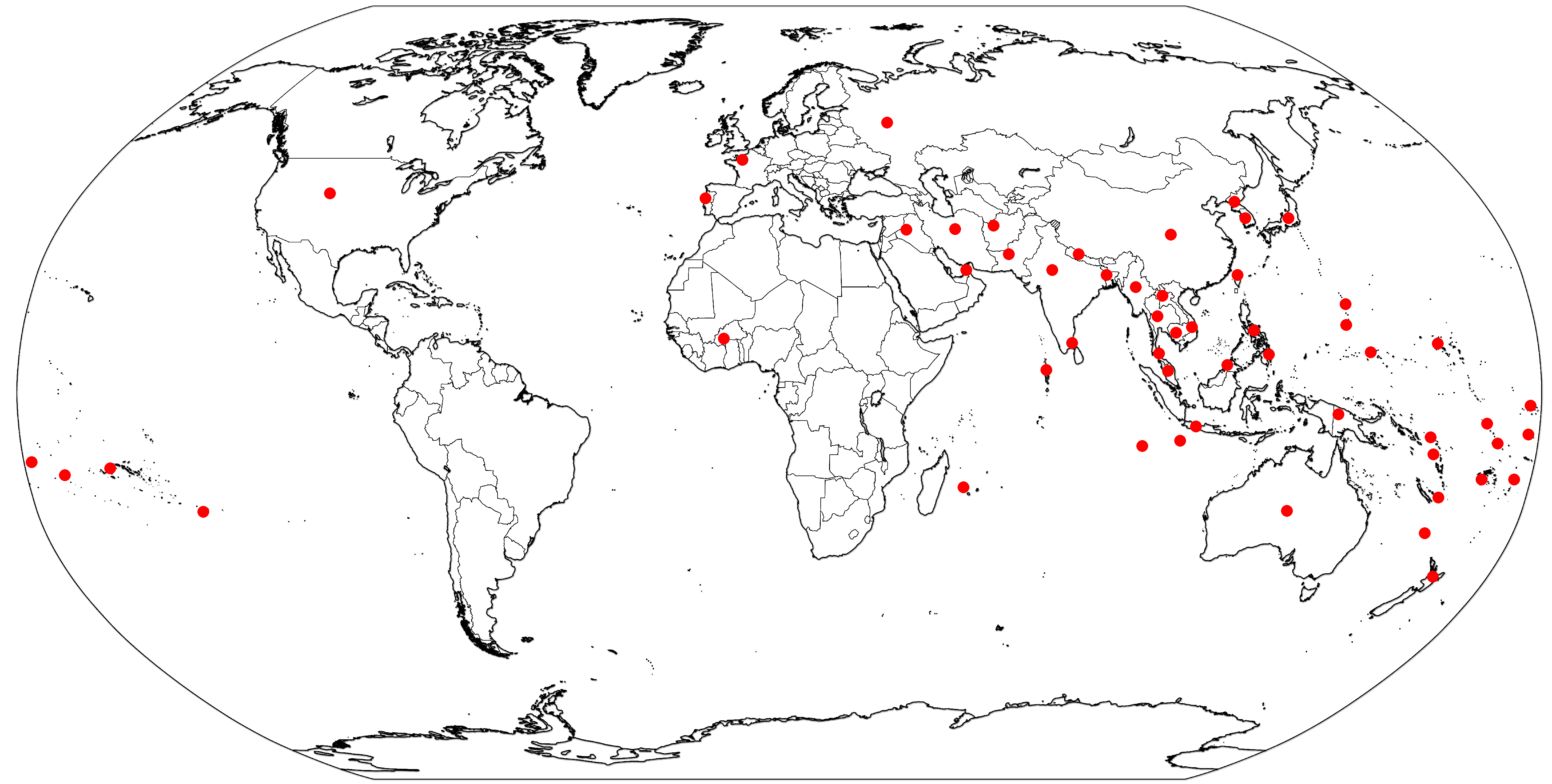


Fig.S1


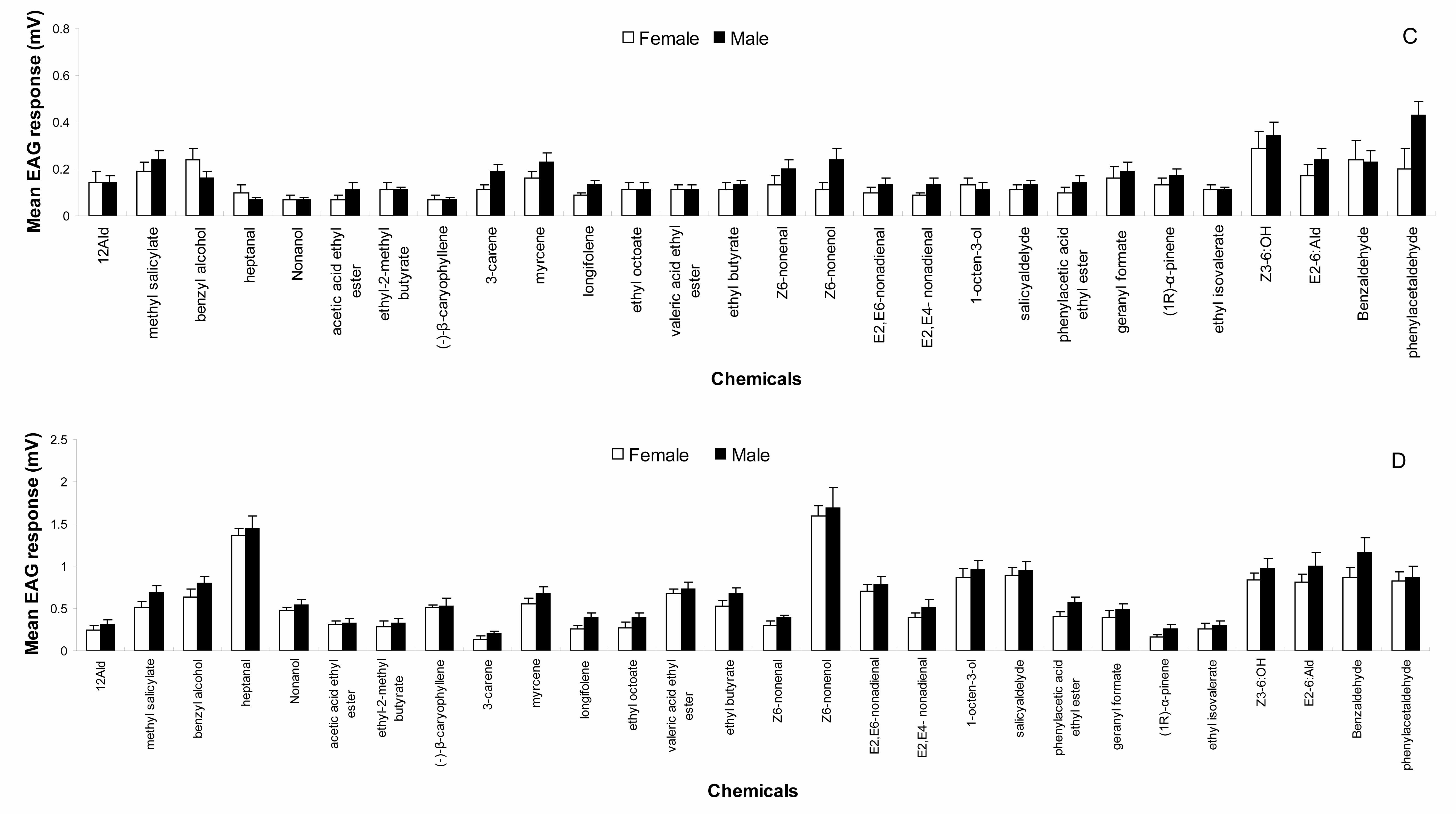


Fig.S2


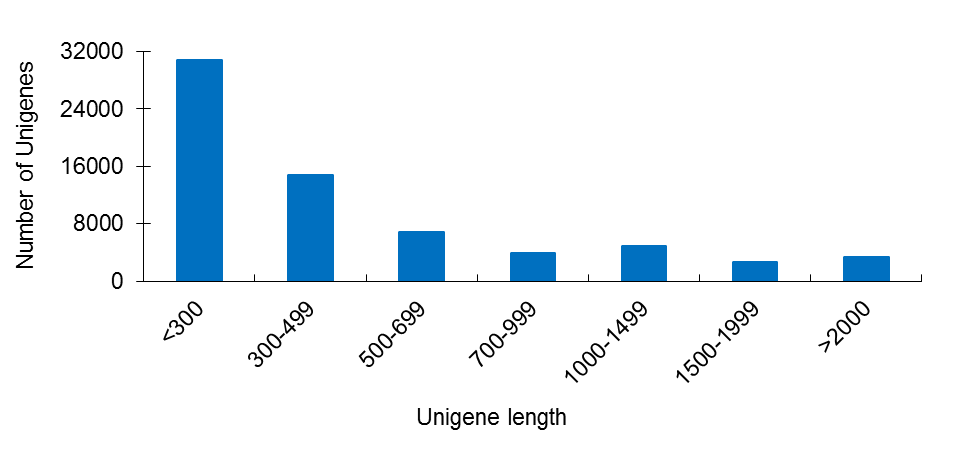


**Fig. S3**


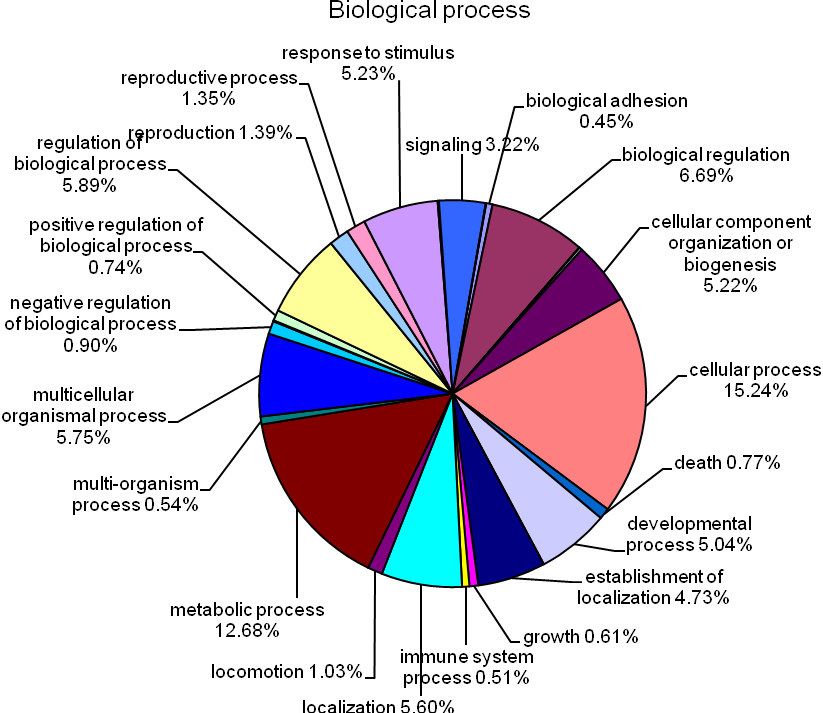


A

B

**Fig. S4**

**Fig. S5**

**Fig. S6**

**Fig. S7**

**Fig. S8**

**Table S1 Chemicals tested in the Electroantennogram recordings**

|  | **Chemicals** | **Purity** | **Sources** |
| --- | --- | --- | --- |
|  | **Insect pheromone compounds and their isomers** |  |  |
| 1 | (9Z,11E)-tetradecadienyl acetate (Z9E11-14:OAc) | 92% | Bedoukian Research Inc |
| 2 | (9Z,12E)-tetradecadienyl acetate (Z9E12-14:OAc) | 93% | Bedoukian Research Inc |
| 3 | 9Z-tetradecenyl acetate (Z9-14:OAc) | 95% | Bedoukian Research Inc |
| 4 | 11Z-tetradecenyl acetate (Z11-14:OAc) | 95% | Bedoukian Research Inc |
| 5 | 9E-tetradecenyl acetate (E9-14:OAc) | 96% | Bedoukian Research Inc |
| 6 | 11E-tetradecenyl acetate (E11-14:OAc) | 98% | Bedoukian Research Inc |
| 7 | 9Z-tetradecen-1-ol (Z9-14:OH) | 95% | Bedoukian Research Inc |
| 8 | 11Z-hexadecenal (Z11-16:Ald) | 95% | Bedoukian Research Inc |
| 9 | 11Z-hexadecenyl acetate (Z11-16:OAc) | 95% | Bedoukian Research Inc |
| 10 | 3-methyl-2-cyclohexenone | 98% | Bedoukian Research Inc |
| 11 | ipsenol | 93% | Bedoukian Research Inc |
| 12 | ipsdienol | 93% | Bedoukian Research Inc |
| 13 | frontalin | 93% | Bedoukian Research Inc |
| 14 | E-β-farnesene | 90% | Bedoukian Research Inc |
|  |  |  |  |
|  | **Green leaf volatiles** |  |  |
| 15 | cis-3-hexen-1-ol (Z3-6:OH) | 98% | Sigma Aldrich Inc. |
| 16 | trans-2-hexenal (E2-6:Ald) | 97% | Sigma Aldrich Inc. |
| 17 | cis-3-hexenyl acetate (Z3-6:OAc) | 99% | Sigma Aldrich Inc. |
| 18 | E2-pentennal (E2-5:Ald) | 95% | Jiaxing Fragrance Inc. |
| 19 | heptanal | 97% | Aladdin Regent database Inc. |
| 20 | nonanal | 97% | Aladdin Regent database Inc. |
| 21 | Z6-nonenal | 97% | Aladdin Regent database Inc. |
| 22 | E2,E6-nonadienal | 93% | Aladdin Regent database Inc. |
| 23 | E2,E4-nonadienal | 93% | Aladdin Regent database Inc. |
| 24 | nonanol | 97% | Aladdin Regent database Inc. |
| 25 | Z6-nonenol | 97% | Aladdin Regent database Inc. |
|  |  |  |  |
|  | **Floral aromatic compounds** |  |  |
| 26 | dodecyl aldehyde (12:Ald) | 97% | Aladdin Regent database Inc. |
| 27 | benzaldehyde | 99% | Sigma Aldrich Inc. |
| 28 | phenylacetaldehyde | 90% | Sigma Aldrich Inc. |
| 29 | benzyl alcohol | 98% | Bodi Chemicals Inc. |
| 30 | 1-octen-3-ol | 97% | Aladdin Regent database Inc. |
| 31 | (1)-linalool | 97% | Sigma Aldrich Inc. |
| 32 | geraniol | 97% | Sigma Aldrich Inc. |
| 33 | isoamyl alcohol | 97% | Aladdin Regent database Inc. |
| 34 | acetic acid ethyl ester | 99% | Aladdin Regent database Inc. |
| 35 | isoamyl acetate | 99% | Aladdin Regent database Inc. |
| 36 | ethyl butyrate | 97% | Aladdin Regent database Inc. |
| 37 | ethyl-2-methyl butyrate | 97% | Aladdin Regent database Inc. |
| 38 | ethyl hexanoate | 97% | Aladdin Regent database Inc. |
| 39 | ethyl heptanoate | 97% | Aladdin Regent database Inc. |
| 40 | ethyl octoate | 99% | Aladdin Regent database Inc. |
| 41 | valeric acid ethyl ester | 98% | Aladdin Regent database Inc. |
| 42 | cis-3-hexenyl butyrate | 97% | Aladdin Regent database Inc. |
| 43 | ethyl isovalerate | 97% | Aladdin Regent database Inc. |
| 44 | salicyaldelyde | 97% | Aladdin Regent database Inc. |
| 45 | methyl salicylate | 98% | Bodi Chemicals Inc. |
| 46 | phenylacetic acid ethyl ester | 99% | Aladdin Regent database Inc. |
| 47 | geranyl formate | 97% | Aladdin Regent database Inc. |
| 48 | allyl Isothiocyanate | 98% | Sigma Aldrich Inc. |
| 49 | γ-unsecalactone | 95% | Jiaxing Fragrance Inc. |
| 50 | acetic acid | 97% | Aladdin Regent database Inc. |
|  |  |  |  |
|  | **Terpenes** |  |  |
| 51 | (-)-β-caryophyllene | 90% | Tokyo Chemical Industry Co., Ltd. |
| 52 | 3-carene | 97% | Sigma Aldrich Inc. |
| 53 | (1S)-α-pinene | 98% | Sigma Aldrich Inc. |
| 54 | (1S)-β-pinene | 98% | Sigma Aldrich Inc. |
| 55 | (1R)-α-pinene | 98% | Sigma Aldrich Inc. |
| 56 | myrcene | 97% | Aladdin Regent database Inc. |
| 57 | longifolene | 97% | Aladdin Regent database Inc. |
| 58 | 1,8-cineole | 95% | Jiaxing Fragrance Inc. |

**Table S2.** Single-end RNA-seq: reads and mapping data

| Sex | Total reads | Total  base pairs | Total  mapped reads | Perfect match | Unique match |
| --- | --- | --- | --- | --- | --- |
| Male | 6,056,792 | 296,782,808 | 5,144,760 | 3,627,773 | 4,199,167 |
| Female | 5,889,529 | 288,586,921 | 5,279,846 | 4,512,285 | 4,315,664 |

**Table S3** Primers used for qPCR.

| Gene | Forward primers (5’-3’) | Reverse primers (5’-3’) |
| --- | --- | --- |
| *ABP1* | CGGAATCACTGAGGAGCAAT | TCTCCAACAGCTTCAACGAA |
| *ABP2* | CTCAAGAAACACCGCACTGA | GGGCCTCATTGTCCATCTTA |
| *ABP3* | CCAATGATATGAAGGCACCA | TGCATTTCGCTGTCCAATAA |
| *ABP4* | CCGAAGAACGATGTGACTGA | CTTGTTTGATCACCGCTTCA |
| *ABPX* | GCGCTGGTAGAGAAGGTCAA | CAGTACAGCCTCGATGTCCA |
| *GOBP1* | ACAAGTGTCGCCAGGAGAGT | GGGGAAGGATTGGATGAACT |
| *GOBP2* | CGTCAAGAGCTTCCCTAACG | TTCAATCATCGCGACTTCTG |
| *OBP1* | GACGTCGATAAAGCCTTGGA | CGGAGATCTCGCTCTTGTGT |
| *OBP2* | CTACCGAAAAGGGCAACAAA | AAACTGTCTCCACGGCATCT |
| *OBP3* | CTTGCTTCATCGCCTGTGTA | CAGCCAGGAGAACACCTCTC |
| *OBP4* | AAGGCGAATACGACATCGAC | GAATCCAAACTTGGGTGCAT |
| *OBP5* | CAGCCTGGTCACTCACTTCA | ACTCTGACGCCGATTCTGTT |
| *OBP6* | TGTGTATCAGTAAACGATGAGTCG | AAAGCTTCTTTCACATCCAGCA |
| *OBP7* | CAAGACGCCAAAGTGTTTCA | TTCCATAGGCTTTCCATTGC |
| *OBP8* | TGTGTGCTGGGTGCAGTTAT | GAGCGCCATATCGAGTTTGT |
| *OBP9* | AGAGCTGTTTATGGCGAGGA | TGACGTCATCTTTCCAATCG |
| *OBP10* | GGAAACAGCACTCGAAATGG | ATGTGTGAGCAGGAATGCAG |
| *OBP11* | CCGAAGAACGATGTGACTGA | TCTCAGCTGGAAACATCACG |
| *PBP1* | CTACTGGCGCGAAGACTACC | GTCGTCCATGAGGTCCAACT |
| *PBP2* | TATCCTCTGCCTCTCCTCCA | CACCTCTCCCACGATGAGTT |
| *PBP3* | TGGAAGAATGCAAGCATGAG | TTCAGCAGCGTGTACTCCAG |
| *CSP1* | GCGTTGGTGTTATGCTGTGT | GCGTATTGGCGCACTATTTT |
| *CSP2* | CCCTACGTCAAGTGCATCCT | ATTTGCCACATTCGTTCTCC |
| *CSP3* | CGACAACGTCAATCTGGATG | CATCGGTACATTTGGCACAG |
| *CSP4* | TACGACAACATCGACCTGGA | GCAGTTGACGTAGGCAGTGA |
| *CSP5* | GCTAAACACCCTGAGGCTTG | TTTTCGGCCAAGAATTTGTC |
| *CSP6* | TGAGTCCCATTACACGGACA | ACACCTTGCGAGTACCATCC |
| *CSP7* | TGTTCTGCTGGTTTTTGCTG | GGCAGCTTCTGTTTCAGTCC |
| *CSP8* | ATGGATCTTGGACCTTGCAC | GCCTTCTCATCTGTGGCTTC |
| *CSP9* | AAGCGGCTGAAGAACGAATA | CTGCGAGTCAAACCTGTTCA |
| *CSP10* | CTCCAACGACAGATTGCTGA | ACGAGGTCTTCCCACTCCTT |
| *CSP11* | TCGTGTTGTCGATTGTGGTT | CAGCGAGGAAGGTTTCGTAG |
| *CSP12* | AAAGGACCTTGCACGAGAGA | AGCGGTCAGGGTTGTATTTG |
| *CSP13* | CACACTCTGCTTCGCTCTTG | CCTTCCACGCTTGTTGGTAT |
| *CSP14* | AATAAGACCCCAGCCCTTTG | TCTCTGCCGTTCAAGAACCT |
| *CSP15* | CGGTTCAGACAGCGTGTAAG | AACGTTTTTCTTGCCAGTCG |
| *CSP16* | GTTCGACTGCTGACTGCGTA | CTCTGGAGTGCAAGGTCCAT |
| *CSP17* | ATGACGGTGGCTTCTACGAC | TTTCAGGGTCGTGCTTCTTT |
| *CSP18* | CGAAGAAAAGGCCACAAGAG | GATGCTTCGAATTTGGGAAA |
| *OR1* | CAGGAAGGTACGCTAGTGGTG | CAGGCAACGACATATCCATT |
| *OR2* | GCCAACACTATCACCGTCCT | CAGTCGAGGGGCTACTTCTG |
| *OR6* | TGGTACTCTCCTTGGGCATC | TCAAAGTCCATGAACGCATC |
| *OR11* | GGCTGGTACGGCACAGTATT | CATCAGGCAGAGGAACAGGT |
| *OR12* | CCGAAAACACGGATGGATAC | TCCCAGAAATGAACCAGACC |
| *OR13* | CGAGAACTTCCCAAAACCAG | TCCAGGAGCAAAAGACATCC |
| *OR14* | GCGTTATTTACCCCTGACGA | CAATGCCAAGAGGTTTCACA |
| *OR17* | ATCAGAATGCCGTTTTGGAG | AACCAGCAAGGAACCATCAC |
| *OR18* | GATTCCCAACGACTTGTACCTC | CCCAGTCAGAATCATCTCCTTC |
| *OR20* | AGCAGTGTCGCATGTTTCAG | TATCGAAGGTCGAACCCGTA |
| *OR22* | CGCTTGGCAGACATAATCAA | AAAGAGCCGACCAACACATT |
| *OR23* | TACGCTAGTGGTGGCACTGA | CATACAGAACCACGTTGAGCA |
| *OR24* | TTACCGTATCCTGCGATTCC | TCCATTTCTTTTTCGCCAAC |
| *OR29* | ACCACTCTCCAGGTTGTTGG | TGTTTGCACCTCTCACGAAG |
| *OR31* | AGTCCGGAGTTTGATGATGC | TCATGTCGAATTGCAGGGTA |
| *OR32* | CATCTGCCAGTTTGATGTGC | CGGAGCGCTTAGTATTGTCC |
| *OR35* | GGCCGCCTACAAGATAGTCA | CAAACGTGTCGAGTGAGAGC |
| *OR36* | ACTGGTTTGCCAATGAGGTC | TGCAACTTCTTGTTCGCTTG |
| *OR41* | CTGCCGCCTGCTTTTACTAC | AGTGGAATCTGGTGCCTTTG |
| *OR43* | TCAGAGAGTTGGTGCAATGG | TGAACAGCGTTGATTTGGAG |
| *OR44* | TTGATCAAATGGCATCAGGA | CACGCTCGAAGTCAACAAAA |
| *OR45* | TGGTTTTCACTCTGGCACTG | AAATCCCTCGCTTCTTCACA |
| *OR48* | CAAGACAGTCGCAGAATGGA | CTGATACGCAATGAGGCAGA |
| *OR53* | TGACGGTACACAGAGCCAAG | CGCCTCCGATAGCTTAGATG |
| *OR55* | ACTGGTGCAACAACGCTTTA | GGGAGAGTCGTACCCGAAGT |
| *OR59* | GCTTTTAGTTTGGCGTCAGC | ATGGGTCGTTTGCATCTTTC |
| *GAPDH* | GGGTATTCTTGACTACAC | CTGGATGTACTTGATGAG |
| *UCCR* | GCCAAGATTGAGATCAAG | GCATACTCCGATAACTAC |

**Reference**

1. Gupta GP, Rani S, Birah A, Raghuraman M: **Improved artificial diet for mass rearing of the tobacco caterpillar, *Spodoptera litura* (Lepidoptera: Noctuidae)**. *Int J Trop Insect Sci* 2005, **25**(01):55-58.

2. Yan FS, Du YJ, Han XL: **A comparative study on the EAG responses of three aphid species to plant volatiles**. *Acta Entomol Sinica* 1994, **1**(1):53–66.

3. Grabherr MG, Haas BJ, Yassour M, Levin JZ, Thompson DA, Amit I, Adiconis X, Fan L, Raychowdhury R, Zeng Q *et al*: **Full-length transcriptome assembly from RNA-Seq data without a reference genome**. *Nat Biotech* 2011, **29**(7):644-652.

4. Iseli C, Jongeneel CV, Bucher P: **ESTScan: a program for detecting, evaluating, and reconstructing potential coding regions in EST sequences**. *Proc Int Conf Intell Syst Mol Biol* 1999:138-148.

5. Conesa A, Gotz S, Garcia-Gomez JM, Terol J, Talon M, Robles M: **Blast2GO: a universal tool for annotation, visualization and analysis in functional genomics research**. *Bioinformatics* 2005, **21**(18):3674-3676.

6. Ye J, Fang L, Zheng H, Zhang Y, Chen J, Zhang Z, Wang J, Li S, Li R, Bolund L: **WEGO: a web tool for plotting GO annotations**. *Nucleic Acids Res* 2006, **34**(suppl 2):W293-297.

7. Li R, Yu C, Li Y, Lam T-W, Yiu S-M, Kristiansen K, Wang J: **SOAP2: an improved ultrafast tool for short read alignment**. *Bioinformatics* 2009, **25**(15):1966-1967.

8. Wang Z, Gerstein M, Snyder M: **RNA-Seq: a revolutionary tool for transcriptomics**. *Nat Rev Genet* 2009, **10**(1):57-63.

9. Mortazavi A, Williams BA, McCue K, Schaeffer L, Wold B: **Mapping and quantifying mammalian transcriptomes by RNA-Seq**. *Nat Methods* 2008, **5**(7):621-628.

10. Audic S, Claverie J-M: **The significance of digital gene expression profiles**. *Genome Res* 1997, **7**(10):986-995.

11. Benjamini Y, Yekutieli D: **The control of the false discovery rate in multiple testing under dependency**. *The Annals of Statistics* 2001, **29**(4):1165-1188.

12. Lu Y, Yuan M, Gao X, Kang T, Zhan S, Wan H, Li J: **Identification and validation of reference genes for gene expression analysis using quantitative PCR in *Spodoptera litura* (Lepidoptera: Noctuidae)**. *PLoS ONE* 2013, **8**(7):e68059.

13. Schmittgen TD, Livak KJ: **Analyzing real-time PCR data by the comparative CT method**. *Nat Protocols* 2008, **3**(6):1101-1108.
